# Transdiagnostic Analysis of Verbal Fluency across Autism Spectrum Disorder, Schizophrenia, and Neurotypical Healthy Control Groups

**DOI:** 10.1192/j.eurpsy.2024.226

**Published:** 2024-08-27

**Authors:** F. Kinga, E. J. Zarka, O. Pesthy

**Affiliations:** ^1^Department of Psychiatry and Psychotherapy, Semmelweis University; ^2^Institute of Psychology, ELTE Eötvös Loránd University, Budapest, Hungary

## Abstract

**Introduction:**

Verbal fluency, a cognitive function that reflects executive functions and the rapid retrieval of pertinent information from memory, has yielded inconsistent findings in previous research on autism spectrum disorder (ASD), however in schizophrenia (SCH) semantic fluency exhibits a more pronounced impairment compared to letter fluency.

**Objectives:**

In this study we aim to comprehensively investigate verbal fluency in ASD, SCH, and neurotypical healthy control individuals (NTP). The primary objective is to investigate disparities in novel response generation, specifically between the ASD, SCH and NTP groups, using phonemic and semantic fluency tasks. Three central inquiries guide our research: (1) whether differences between groups (ASD, SCH, and NTP) can be identified in word productivity, clustering, errors, and perseverations; (2) whether participants with ASD and SCH exhibit different word production with elevated imageability and concreteness values; and (3) if individuals with ASD and schizophrenia demonstrate reduced productivity during the earlier phases of fluency tasks.

**Methods:**

Forty participants with ASD (12 female, 24 male, 4 other, mean age: 30.5), 39 with SCH (10 female, 28 male, 1 other, mean age: 34.7) and 41 NTP (13 female, 28 male, mean age: 31.0) were recruited from the outpatient units of the Department of Psychiatry and Psychotherapy, Semmelweis University. Participants were requested to list as many words as they could on two phonemic and two semantic category conditions. Audio recordings were later transcribed. To assess concreteness and imageability, we employed a seven-point scale and recruited independent external raters to evaluate a total of 1481 words.

**Results:**

Preliminary results indicate that the three study groups did not differ significantly in phonemic fluency (*F*(2, 119)= 0.983, *p*=0.377), during either time period. However, a significant difference was observed in semantic fluency (*F*(2, 119)= 6.531, *p*=0.002). Post-hoc tests (Tukey corrected) revealed that this difference stemmed from impaired performance in the SCH group. Participants with schizophrenia (SCH) exhibited reduced semantic word productivity compared to both neurotypical (NTP) individuals and participants with ASD (Figure 1). However, there were no significant differences between participants with ASD and NTP individuals.

**Image:**

**Figure:**
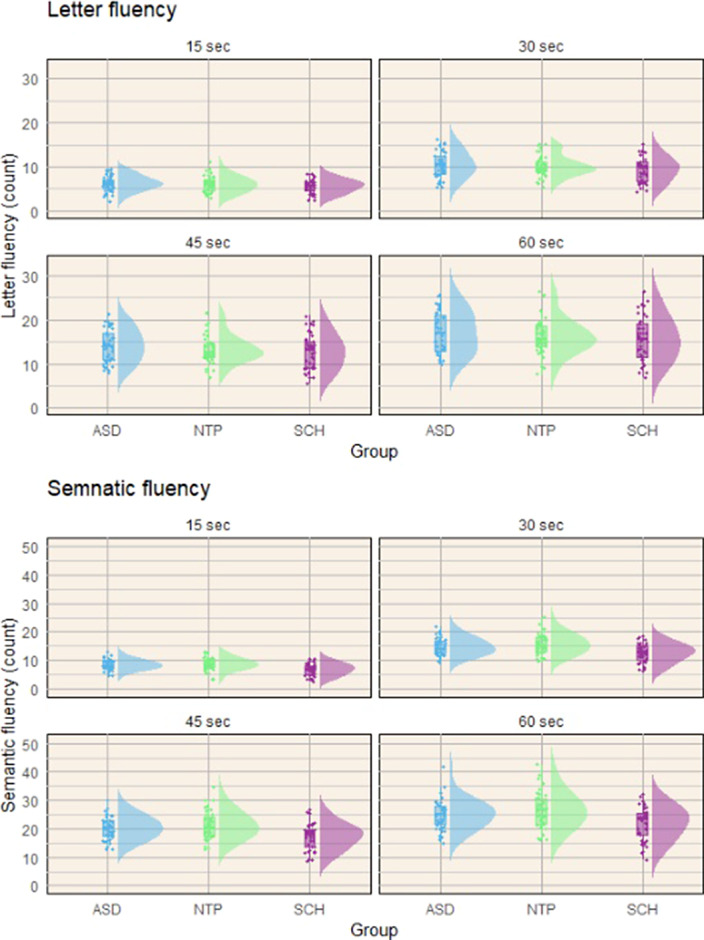

**Conclusions:**

In conclusion, our study investigated the characteristics of verbal fluency in a transdiagnostic approach. While phonemic fluency did not reveal significant differences among the three groups, our analysis of semantic fluency unveiled a distinction. Specifically, individuals with schizophrenia exhibited impaired semantic word productivity. Our study highlights the complex nature of verbal fluency impairments in different conditions and the importance of considering more nuanced methods when assessing cognitive functions.

**Disclosure of Interest:**

None Declared

